# Hepatitis B control in jeopardy

**DOI:** 10.1007/s10096-026-05542-0

**Published:** 2026-05-12

**Authors:** Efstathios Karakasidis, Rafael Mazin

**Affiliations:** 1 Faculty of Medicine, School of Health Sciences, University of Thessaly, Larissa, Greece; 2 Former senior advisor on viral hepatitis, HIV and STIs of PAHO/WHO, Washington, US

## Abstract

This paper analyzes the impact of delaying the Hepatitis B vaccine beyond the first day of life following the recently updated guidelines of the United States Centers for Disease Control and Prevention (CDC) regarding universal neonatal vaccination. We discuss the potential effects that delaying the initial hepatitis B vaccine dose until 2 months will have on global health communities, the economy, well-being, and development. Despite the WHO 2030 elimination goals, shifts toward delaying the newborn “birth dose” and rising vaccine hesitancy pose a risk of a public health reversal resulting in hepatitis B (HBV) remaining a global threat. We argue that maintaining universal birth-dose recommendation should remain the most reliable strategy to mitigate systemic screening gaps and achieve the global elimination of hepatitis B, along with other forms of viral hepatitis.

## Text

Recently, discourse surrounding the United States CDC recommendations has introduced potential ambiguity regarding the universal newborn hepatitis B vaccine dose. While the guidelines acknowledge a birth dose, they allow for a delay until two months of age for infants born to mothers who test negative for the virus [1]; however, recent judicial intervention has halted these changes. A federal judge recently issued a stay on these new recommendations, effectively pausing their implementation due to concerns over procedural adherence and public health safety [2]. The CDC recommendation may be based on previous questionable statements, like that about the presumed association of vaccination and autism [3] and reflects an effort to accommodate parental preferences or to address broader vaccine hesitancy. The purported link between vaccinations and autism originated from a now-retracted study by Wakefield et al., which suggested a correlation between the Measles, Mumps, and Rubella (MMR) vaccine and the onset of autism [4]. Despite the subsequent retraction and the scientific community’s inability to replicate the findings, the initial publication sparked widespread public concern. The scientific consensus, reinforced by a meta-analysis of over 1.2 million children by Taylor et al., confirms that no such association exists [5]. Furthermore, sociopolitical arguments that categorize HBV as a “lifestyle disease” restricted to specific adult behaviors ignore the high risk of horizontal transmission within households and the extreme vulnerability of the neonatal immune system.

While a functional immune system is typically capable of the complete clearance of hepatitis A (HAV), particularly in individuals within endemic regions who have access to vaccination [6], hepatitis C (HCV) has only recently become curable through advanced pharmacological interventions that mitigate chronic liver disease and irreversible hepatic damage [7]. In contrast, a definitive cure for HBV remains elusive. Management of HBV currently relies on a highly immunogenic vaccine that provides robust prophylaxis, achieving serological protection levels exceeding 95% in healthy populations and effectively preventing viral acquisition [8]. Vaccination provides both direct and indirect protection, known as herd immunity. By ensuring that a high threshold of the population is immunized, the probability of pathogen transmission is substantially marginalized. This collective immunity not only mitigates the overall epidemiological burden but also provides a pathway toward the potential eradication of the disease [9]. The impact of the hepatitis B vaccine is clearly demonstrated in Guangdong, where rising vaccination coverage-from an already high 98.56% in 2005 to near-universal levels of 99.60% by 2022**-** has substantially reduced the incidence of both acute and chronic HBV. Specifically, reported acute cases fell from 7,509 in 2005 to 2,097 in 2022, while among the most vulnerable age group (0–1 years), chronic HBV cases declined from 37 in 2005 to 10 in 2022. This dramatic reduction highlights the vaccine’s efficacy, particularly as children and adolescents have a very high susceptibility to chronic infection [10]. Taiwan serves as an example of the success of early intervention. By implementing a targeted immunization strategy in July 1984 for infants born to HBsAg-positive mothers, the nation met the WHO 2020 goals three years ahead of schedule in 2017 [11]. The impact is most evident when comparing age cohorts: while adults aged 30 and older (born before the vaccine era) maintain an HBsAg positivity rate of 6.7%, the prevalence among children under 9 years old dropped to just 0.2% by 2014 [11]. This evidence informs the WHO Global Health Sector Strategy on viral hepatitis 2016–2021, which states that birth-dose vaccination against the hepatitis B virus (HBV) is essential for preventing hepatitis in infants and for eliminating this scourge. This WHO recommendation is based on a strategy to attain 90% reduction in new cases and a 65% reduction in hepatitis-related deaths by 2030, with 2015 as reference [12].

HBV infection is a global issue, as in 2022, 254 million people had chronic HBV infection, with 1.2 million new infections each year [13]. It is asymptomatic in the majority of patients, but 1.1 million deaths are attributed to hepatitis B complications such as cirrhosis and hepatocellular carcinoma (HCC) [11, 14, 15]. HBV can be significantly transmitted perinatally or vertically from mothers who are HBsAg positive to newborns, through unsafe injection and medical procedures, and less commonly via sexual contact [11, 12, 16].

Both the American College of Obstetricians and Gynecologists (ACOG) and CDC suggest that in pregnant women, triple-panel testing (HBsAg, anti-HBs, and total anti-HBc) be performed in the first trimester if not previously documented or known to them, and regardless of vaccination status [15, 17]. The shift toward allowing parental discretion to determine HBV vaccination timing is a harmful regression in public health policy, given the fact that in the United States, only 33.9% of persons with HBV infection and 11.7% of those past exposed to HBV were aware of their status [18]. On top of this, a study found that 14% of pregnant women who gave birth each year were not tested for HBsAg to prevent perinatal transmission. Furthermore, over 50% of HBsAg-positive individuals do not receive recommended monitoring during or after pregnancy [19]. Relying on maternal screening is further complicated by occult HBV (HBsAg-negative, HBV-DNA positive); perinatal transmission has been documented from mothers who test negative for HBsAg but possess low-level viremia associated with anti-HBc seropositivity [20].

Early immunization (within the first 24 h after birth), as recommended by the World Health Organization, has become a common practice in many countries [21]. This approach seems to be very advantageous because it has no contradictions, and the side effects are mild and transient (fever, fatigue, swelling at the injection site etc.). Beyond preventing vertical transmission, this strategy protects newborns from potential risks associated with horizontal transmission via medical instruments or piercing procedures throughout childhood, adolescence, and adulthood [21]. Above all, universal vaccination would reduce the burden of hepatitis B in the population and would permit focusing a large amount of public health efforts on the control of other viral hepatitis (hepatitis A, C, D, and E).

Conversely, the dangers of declining vaccination rates are evidenced by the recent measles resurgence in the Americas, where a 31-fold increase in cases was documented in 2024. Notably, 92% of the 1,454 confirmed cases in the United States occurred in individuals who were unvaccinated or had unknown vaccination histories, illustrating how quickly public health gains can be reversed [22].

An increase in both acute and chronic HBV cases will impose a significant burden on global health systems and economic stability. Direct expenditures are expected to rise sharply due to the increased necessity for diagnostic testing, prolonged medication regimens, and the intensive long-term management of chronic infections. These costs are compounded by significant indirect economic impacts, including productivity losses attributable to workplace absenteeism, long-term disability, and premature mortality [23]. A cost analysis in China, which accounts for 30% of the global HBV burden, concluded in 2020 that HBV vaccination is projected to save approximately $120.1 billion US dollars due to reduced future healthcare costs, improved quality of life, and reduced lost productivity [24]. Furthermore, a higher prevalence of chronic HBV is correlated with increased mortality rates stemming from severe complications such as cirrhosis and hepatocellular carcinoma. The clinical significance of HBV in pediatric populations lies primarily in the high rate of progression to chronic infection during the first months of life. While acute HBV is often asymptomatic in younger patients, the probability of developing a chronic carrier state is approximately 90% in infants and 30% to 50% in children aged one to five, compared to only 5% in those infected as adults [25]. This high rate of chronicity significantly increases the lifetime risk of developing cirrhosis and hepatocellular carcinoma in later adulthood.

Furthermore, the suppression of HBV is the primary mechanism for controlling Hepatitis D (HDV), which requires the HBsAg envelope for replication. An increase in the HBV reservoir inevitably expands the biological substrate for HDV transmission. In the general population, the global estimated anti-HDV prevalence is 4.5% among HBsAg-positive people, with coinfection rates having heterogeneous geographic distribution ranging from 3.0% in Europe to 6.0% in Africa [26]. Epidemiological data from Italy confirm that the decline in HDV incidence has occurred in direct parallel with robust HBV vaccination programs [27]. This can be explained by the fact that the vaccine also controlled HDV by decreasing the pool of HBV chronic carriers, who represent the biological substrate for HDV spreading [28].

## Conclusion

The clinical window for preventing HBV chronicity is narrow; the risk of a lifelong carrier state is 90% for infants infected at birth versus 5% for those infected as adults. Any policy that introduces optionality or delay into the birth-dose schedule risks a resurgence of pediatric infections that will haunt public health systems for decades. The CDC must reaffirm a rigid, universal birth-dose mandate to ensure the WHO 2030 elimination targets remain achievable.

## Data Availability

No datasets were generated or analysed during the current study.
